# A hybrid deep learning framework combining CTGAN and transformers for personalized injury mitigation in elite sports

**DOI:** 10.3389/fphys.2026.1923556

**Published:** 2026-10-07

**Authors:** Tiemin Teng, Lei Zhang

**Affiliations:** 1 School of Physical Education, Harbin Normal University, Harbin, Heilongjiang, China; 2 Education College, Wenzhou Business College, Wenzhou, China

**Keywords:** athlete performance, CTGAN, deep learning, injury risk, personalized training, sports analytics, sports injury prediction, transformer models

## Abstract

**Introduction:**

Optimizing training in elite sports while managing injury risk requires models that can learn from heterogeneous and imbalanced athlete records without contaminating evaluation data. This study presents PTI-GANFormer (Personalized Training and Injury Risk Prediction using Generative Adversarial Network-Former), an integrated decision-support framework. Its contribution is not CTGAN or self-attention in isolation; rather, it is the leakage-controlled combination of training-only conditional augmentation, three functionally separated attention pathways, five domain-engineered indicators, and a transparent risk-to-action recommendation layer.

**Methods:**

After median imputation, plausibility-aware outlier handling, categorical embedding, scaling, and feature engineering, the data were stratified into training and untouched testing partitions. CTGAN was fitted only to the training partition and generated minority-class records until each training class matched the original majority count of 7,894. The hybrid classifier distinguishes No Injury, Minor Injury, and Major Injury, while the recommendation engine converts predicted risk into advisory workload and recovery options that require practitioner review.

**Results:**

Across repeated stratified evaluation runs, PTI-GANFormer achieved an accuracy of 0.960 ± 0.004 (95% CI: 0.957–0.963), precision of 0.950 ± 0.006, recall of 0.960 ± 0.005, F1-score of 0.950 ± 0.005, and macro ROC-AUC of 0.981 ± 0.004.

**Discussion:**

These results support predictive risk stratification and decision support; they do not by themselves establish injury prevention or performance enhancement.

## 1 Introduction

Elite sportspeople face high physical and psychological loads, which can put them at risk of injury and deterioration in their performance. As wearable sensors and physiological monitoring technology evolve alongside the rise of sports analytics and performance platforms, there has been an opportunity to leverage data for athlete management. The last few years have seen tremendous success in deep learning when it comes to pattern learning from temporal sports-related data and predicting injury occurrences in the future (Ye et al., 2023). In recent years, realtime monitoring technologies have grown in significance to monitor athlete health and performance. Injury monitoring systems involving deep learning can aid in realtime assessment of physiological and biomechanical parameters for timely interventions and preventive measures (Ren et al., 2024). In addition, the advent of Generative Adversarial Networks (GANs) has provided sports applications with new opportunities to create realistic synthetic data and improve the training of models when data is scarce (Eerdenisuyila et al., 2024).

The accurate prediction of sports injuries and the optimization of their recovery is still a big challenge in the world of elite sports. The use of machine learning techniques to estimate the risk of reinjury and inform individual rehabilitation planning from physiological testing data has been demonstrated to work well (Abasi et al., 2025). Furthermore, multimodal sensor fusion systems have been effective in acquiring and fusing various athlete health parameters, allowing for more comprehensive monitoring systems (Gai, 2025). In recent years, machine learning frameworks have also shown that risks of injuries can be predicted while at the same time optimizing athletic performance by using intelligent decision models (Raju et al., 2026). Conventional workload monitoring techniques are based on fixed levels of thresholds that may not fully reflect the dynamic nature of athlete adaptation. In order to model complex workload variations, advanced temporal learning frameworks have been developed, and therefore the prediction of injury risk using such models has been enhanced (Pan et al., 2026). In addition, time-to-injury forecasting methods have received attention as methods of predicting when injuries will occur, enabling coaches and medical staff to take proactive measures to prevent injuries from occurring (Catterall, 2026).

In elite sports, the utilization of decision-support systems with machine learning has been gaining traction, with the aim of enhancing injury management practices. The systems combine past injury data, training loads, and physiological data to assist in decision-making processes (Huth et al., 2025). Research examining lifestyle, recovery, and workload factors has revealed that the onset of injury is often a result of a multitude of interacting factors, rather than a single event (Rossi and Rodrigues, 2025). Visualization techniques have also contributed to the intelligibility of injury prediction models, which allows sports practitioners to understand the factors that contribute to injuries (Lee et al., 2025). A major issue in developing predictive models is the limited availability of injury data. However, the problem of limited data has been addressed by a novel data generation method that generates realistic athlete profiles to enhance the generalization of the model (Cordeiro et al., 2025). There are several machine learning injury prediction models that have shown promising results in identifying athletes at high risk prior to injury (Iduh et al., 2024; Xu et al., 2025). Furthermore, with the help of the Internet of Things (IoT) technology, realtime athlete data can be gathered continuously, which is a solid base for the development of intelligent sports analysis applications (Wu et al., 2023).

These developments have thus far been mostly based on the principle of either injury prediction or performance optimization, separately. The need is for an integrated framework to optimize personalized training approaches as well as reduce injury risk. To tackle this problem, in this study we describe a framework based on a Generative Adversarial Network which takes advantage of the multimodal nature of an athlete’s data to generate personalized training plans, and of the proactive identification of potential injuries. The proposed solution is designed to optimize the performance of athletes, facilitate personalized training adaptation, and optimize long-term management of athlete health in elite sports settings.

### 1.1 Main contribution

The main contributions of this work are as follows:

- PTI-GANFormer integrates leakage-controlled CTGAN augmentation, complementary attention pathways, domain feature engineering, and advisory training-load mapping within one reproducible injury-risk decision-support pipeline.
- CTGAN is used as an established training-only class-balancing tool; it is not claimed as a new generative algorithm.
- Five domain-engineered indicators-Recovery Index, Fatigue Ratio, Fatigue Level, Biomechanical Strain Index, and Training Stress Index-are evaluated individually through sensitivity analysis.
- Three attention branches are assigned non-identical roles: within-record feature interaction, batch-level athlete similarity, and hierarchical contextual tabular representation; component-wise ablations test whether each role adds measurable value.
- A transparent recommendation engine maps calibrated risk levels to conservative workload and recovery options and is empirically compared with fixed-threshold and unconstrained alternatives.
- The framework predicts injury-risk categories and provides practitioner-facing recommendations; prospective intervention studies are required before claims of injury prevention or performance improvement can be made.

## 2 Literature review

Shi et al. (2025) suggested a deep learning model for predicting athlete injuries based on wearable sensor data, which was a time-series forecasting framework. The model was used to analyze changes in physiological and movement-related parameters and to identify potential injury risks. The experimental results showed that the model could be used to predict the occurrence of injuries more effectively, capturing sequential patterns that indicate injury risk. The research showcased the benefits of wearable sensing for ongoing monitoring of athletes. But the performance of prediction was affected by the quality of the sensors and the availability of consistent longitudinal data. Hu et al. (2025) created an Internet of Things (IoT) based deep learning monitoring system that enables realtime assessment and feedback generation of athletes’ performances. The system was designed to combine wearable technology, cloud computing, and intelligent data analysis to provide realtime data on performance. The outcomes indicated successful realtime monitoring and fast feedback, which enabled making informed decisions about training. Although the framework worked well in the performance assessment, it lacked an emphasis on the long-term assessment of injury risk.

Alzahrani et al. (2026) proposed a new wearable biomechanics approach to realtime optimization of sports injury prevention and rehabilitation. The system was based on a fusion of biomechanical sensing and machine learning techniques to identify abnormal movement patterns linked to the development of injury. Findings from experiments showed good identification of injuries and improved monitoring of rehabilitation. The framework relied on the use of specialized wearable devices, however, which could impact the scalability of the framework in various sporting contexts. In a recent study, Alanazi et al. (2026) developed an adaptive ensemble learning (AEL) approach that combines reinforcement learning for realtime ankle injury detection. The suggested approach automatically modified model parameters based on the varying characteristics of the sensor data. Results showed that the accuracy of detection and adaptability were improved compared to the conventional ensemble models. However, the complexity of the reinforcement learning algorithm could make realtime implementation a costly operation.

The machine learning approaches to predict musculoskeletal loading at common running injury locations using instrumented insoles were explored by Van Hooren et al. (2024). The study quantified the biomechanical stress associated with injury and showed good prediction of the risk of injury related to loading conditions. With the results, the need for insole-based sensing systems for injury prevention was confirmed. The study was, however, limited to injuries relating to running. Wu et al. (2026) proposed a machine learning (ML) approach involving multiple disciplines to predict running injuries, including physiological, biomechanical, and training information. Using multiple factors related to the athlete, the model also performed better in predicting performance. The study showed that analyzing multiple data sources can be useful in improving the injury risk assessment. The ability to gather and store complete data sets of athletes, though, is still problematic in the real world.

To solve the problem of sports injury prediction, Fang and Chen (2025) presented a temporal graph encoding and graph neural network sports injury prediction model that is capable of cross-sport transfer learning. The model reflected the complex relationships between training activities, athlete characteristics, and injury events. The experimental results demonstrated the effectiveness of transfer learning for different sports domains. But there was a problem: the framework needed vast amounts of data and a good amount of computing power to work effectively. Hu et al. (2026) proposed a hybrid model for athletic injury risk prediction that incorporates features and attention mechanisms. The framework employed attention mechanisms to detect key features that are relevant to injuries, while enhancing predictive accuracy. Results showed that prediction accuracy was higher and feature representation was better than that of the conventional machine learning approaches. However, the experience of the model architecture made the decision-making process less transparent.

Wang (2025) created a model called ‘Improved ARIMA’ to forecast the likelihood of injuries occurring in digital physical education settings. The framework used injury trends and athlete activity data from the past to predict future injury risks. Experimental results showed that the forecasting for temporal injuries was satisfactory. The statistical model, however, had some limitations, as it could not capture highly nonlinear relationships as those that exist in sport data. Ma et al. (2025) presented an interpretable machine learning model for predicting injuries in university football players using a SHAP-based approach. The model implemented a predictive analytics approach with explainable AI methods to uncover influential injury-related factors. Results showed injury classification was correct, and clear explanations for prediction results were given. The framework, however, has been tested with university-level athletes, which may restrict its applicability to the very best in sport. A summary of recent studies in the field of athlete injury prediction and performance monitoring can be seen in **Table 1**.

**Table 1:** Comparative analysis of recent machine learning and deep learning approaches for athlete injury prediction, prevention, and performance monitoring.

Author, ref & year	Methodology	Application area	Key findings	Strengths	Limitations
Shi et al. (2025)	Deep learning with time-series forecasting using wearable sensor data	Athlete injury prediction	Effectively identified temporal injury patterns from sensor data	Captures sequential athlete behavior and injury trends	Sensitive to sensor noise and data quality
Hu et al. (2025)	IoT-enabled deep learning monitoring system	Real-time performance monitoring	Enabled continuous athlete tracking and instant feedback	Supports real-time decision making	Limited focus on long-term injury prediction
Alzahrani et al. (2026)	Wearable biomechanics framework with machine learning	Injury prevention and rehabilitation	Detected abnormal movement patterns associated with injuries	Integrates injury prevention and rehabilitation monitoring	Requires specialized wearable devices
Alanazi et al. (2026)	Reinforcement learning-based adaptive ensemble model	Real-time ankle injury detection	Improved injury detection accuracy in dynamic environments	Adaptive learning improves detection performance	High computational complexity
Van Hooren et al. (2024)	Machine learning with instrumented insoles	Running injury assessment	Predicted musculoskeletal loading at injury-prone locations	Uses practical and wearable sensing technology	Applicable mainly to running-related injuries
Wu et al. (2026)	Multidisciplinary machine learning framework	Running injury prediction	Improved prediction using physiological and biomechanical data	Considers multiple injury-related factors	Requires extensive multidimensional datasets
Fang and Chen (2025)	Graph Neural Networks with transfer learning	Cross-sport injury prediction	Enhanced prediction across different sports disciplines	Enables knowledge transfer between sports	Needs large datasets and computational resources
Hu et al. (2026)	Attention-based hybrid deep learning model	Athletic injury risk prediction	Improved feature extraction and prediction accuracy	Identifies important injury-related features automatically	Reduced interpretability due to model complexity
Wang (2025)	Improved ARIMA forecasting model	Sports injury probability prediction	Achieved effective temporal injury forecasting	Simple and computationally efficient	Limited capability for nonlinear data modelling
Ma et al. (2025)	SHAP-based interpretable machine learning	Football injury risk prediction	Provided accurate and explainable injury predictions	Enhances model transparency and interpretability	Evaluated mainly on university athletes

### 2.1 Research gaps

Previous studies have established that machine learning, deep learning, wearable sensing, IoT monitoring, synthetic-data generation, and attention mechanisms can support athletic risk assessment. Therefore, this study does not claim CTGAN or Transformer attention as stand-alone algorithmic innovations. The narrower contribution is a leakage-controlled integration in which training-only conditional augmentation is paired with three explicitly separated representation pathways and a transparent recommendation layer.

A recent calibration and risk-model evaluation study (Rahman et al., 2026), together with broader prediction-model assessment literature (Van Calster et al., 2016), emphasizes that contemporary risk models should not be judged by accuracy alone. Instead, they should be evaluated using comparative discrimination, uncertainty estimation, calibration, and operational interpretability. This perspective is particularly relevant for injury-risk decision support, where clinicians and coaches require not only accurate predictions but also reliable and actionable outputs.

The applicability of the individual components used in the proposed framework is also supported by prior work. Conditional tabular GANs have been widely used for modeling mixed-type and imbalanced tabular data, demonstrating their usefulness for synthetic record generation and class balancing (Xu and Veeramachaneni, 2019). Transformer-based tabular models, including FT-Transformer (Gorishniy et al., 2021), TabTransformer (Huang et al., 2020), and SAINT (Somepalli et al., 2021), have shown that feature-token self-attention and inter-sample attention can improve representation learning on heterogeneous tabular datasets. In addition, temporal fusion transformers have demonstrated the value of attention-based architectures for interpretable multi-horizon forecasting, which is relevant to athlete monitoring and workload-related risk assessment (Lim et al., 2021).

In sports injury analytics, synthetic-data approaches, graph-based models, and attention-based deep learning methods have been applied to injury-risk prediction and athlete monitoring (Cordeiro et al., 2025; Fang and Chen, 2025; Hu et al., 2026). These studies demonstrate the potential of advanced machine learning methods in sports-related risk prediction. However, most prior works evaluate augmentation, attention mechanisms, or predictive performance in isolation. Few studies provide a leakage-controlled pipeline in which training-only synthetic augmentation, separated attention pathways, domain-engineered indicators, and a constrained recommendation layer are jointly evaluated on the same untouched test partition.

Relative to such risk-assessment work, PTI-GANFormer is positioned as an integrated tabular decision-support pipeline and is evaluated through same-split baselines, confidence intervals, paired significance tests, low-level ablations, and engineered-feature sensitivity analysis.

## 3 Proposed PTI-GANFormer framework

The proposed PTI-GANFormer framework predicts injury-risk categories and produces advisory, practitioner-reviewed training recommendations. The multimodal dataset contains 15,420 records and 29 raw attributes. Missing numeric values are median-imputed using parameters learned from the training partition; duplicates are removed before splitting; and IQR limits are used to flag potential anomalies rather than automatically delete physiologically plausible high-load events. Only demonstrably impossible sensor values are winsorized to domain-valid bounds. Categorical variables are mapped to learned embeddings, avoiding an artificial ordinal hierarchy. Five domain-specific indicators-Recovery Index, Fatigue Ratio, Fatigue Level, Biomechanical Strain Index, and Training Stress Index-expand the predictor set from 29 to 34 features. A stratified 80:20 split is performed before any scaling or synthesis. The scaler and CTGAN are fitted exclusively on the training partition, while the 3,084-record test set remains unchanged. Three complementary attention pathways then classify No Injury, Minor Injury, and Major Injury, and the recommendation engine maps calibrated risk to conservative workload, recovery, sleep, and physiotherapy options. **Figure 1** summarizes this leakage-controlled workflow.

**Figure 1:**
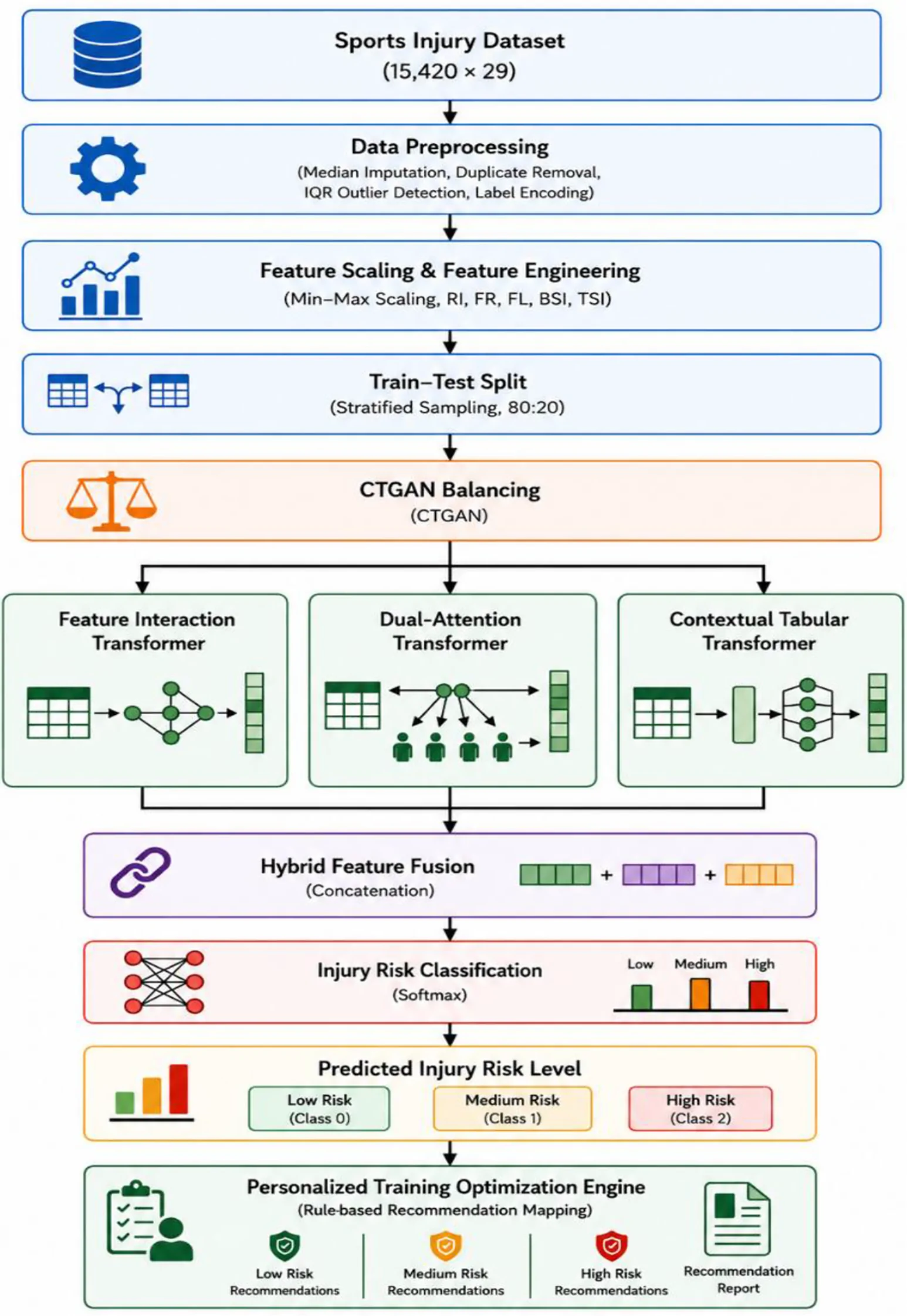
Overall workflow architecture of the proposed PTI-GANFormer framework for personalized training optimization and injury risk prediction in elite athletes.

### 3.1 Data collection

Prediction of injury risk and training optimization is contingent upon having complete data related to the athletes. Hence, a multidimensional sports injury dataset was gathered, which contained information relevant to athlete performance, their recovery state, training load, and injuries sustained by the athletes. Data for analysis were collected from the Kaggle repository at https://www.kaggle.com/datasets/anjalibhegam/multimodal-sports-injury-dataset, which has 15,420 observations and 29 attributes. The attributes contain critical data related to the physical condition and training routines of the athletes, which can help develop an effective injury risk prediction model.

The collected dataset is expressed as in Equation 1, where D refers to complete dataset, N refers to overall athlete records, $x_{i}$ refers to feature vector of the *i-*th athlete and $y_{i}$ refers to corresponding injury risk label. In this work, the dataset contains N=15420samples.

(1) $$D={\left\{(x_{i},y_{i})\right\}}_{i=1}^{N}$$

The input feature matrix is represented as in Equation 2, where X contains overall athlete-related attributes used for the analysis of injury risk. The feature vectors contain 29 variables related to various aspects of athletic training, recovery, and physiological state.

(2) $$X=[x_{1},x_{2},\ldots ,x_{N}]$$

The target variable is expressed as in Equation 3, where Y refers to the injury outcome associated with each athlete record.

(3) $$Y=\{y_{1},y_{2},\ldots ,y_{N}\}$$

The obtained dataset acts as the basis for performing preprocessing, feature engineering, data balancing, and hybrid transformer-based injury risk predictions using the proposed PTI-GANFormer architecture.

### 3.2 Data preprocessing

Raw datasets used for sports usually have missing values, duplicates, outliers, and categorical features that can impair the learning process and lead to ineffective predictions. Hence, a full-scale pre-processing is carried out before analyzing injury risk and training models. The pre-processing step involves the treatment of missing values, duplicate removal, outlier detection, and encoding categorical features.

For missing data, median imputation is carried out. This technique retains the central trend of the data while minimizing the impact of outliers. The imputation equation for a variable is provided by Equation 4, where $x_{ij}$ refers to value of the *j-*th feature for the *i*-th feature record and $\text{Median }({\text{X}}_{\text{j}})$ refers to median value of the corresponding feature column.

(4) $$x_{ij}=\{\begin{array}{cc} x_{ij}, & \text{if }x_{ij}\neq \text{NULL}\\ \text{Median}(X_{j}), & \text{if }x_{ij}=\text{NULL} \end{array}$$

Duplicating data is removed to avoid duplicate data and minimize any sort of bias while training the model. The original dataset will be denoted as D. The duplicate-free dataset is achieved as in Equation 5, where $D_{unique}$ refers to the cleaned dataset after removing all repeated records.

(5) $${\text{D}}_{\text{nodup}}=\text{ D }-{\text{ D}}_{\text{duplicate}}$$

The IQR rule is used as a diagnostic flag, not as a generic deletion rule. For each continuous feature, the lower and upper screening limits are defined in Equations 6–8. Observations outside these limits are retained when they represent physiologically plausible high-load or fatigue events, because such extremes may be clinically informative injury triggers. Only values that violate predefined measurement plausibility (for example, impossible negative duration or sensor values outside the device range) are winsorized to the nearest valid boundary. All thresholds are estimated from the training partition and then applied unchanged to validation and test data.

(6) $$\text{IQR }={\text{ Q}}_{3}-{\text{ Q}}_{1}$$

(7) $$\text{LB }={\text{ Q}}_{1}-\;1.5\;\times \text{ IQR}$$

(8) $$\text{UB }={\text{ Q}}_{3}+\;1.5\;\times \text{ IQR}$$

Categorical variables are not assigned linear label codes. Each category is represented by a learned embedding vector, as formalized in Equation 9, so nominal categories enter the attention layers without an imposed ordinal distance. Unknown test-time categories are mapped to a dedicated out-of-vocabulary token, and the vocabulary is learned from training data only.

(9) $$\text{e}({\text{c}}_{\text{j}})={\text{ E}}_{\text{j}[\text{k}({\text{c}}_{\text{j}})]},\text{ k}({\text{c}}_{\text{j}})\in \{0,\;\ldots ,{\text{ K}}_{\text{j}}\}$$

Here, $E_{j}\in {\mathbb{R}}^{K_{j}\times d_{emb}}$ denotes the learnable embedding matrix for the j-th categorical feature, where $d_{emb}$ is the embedding dimension. The function $k(c_{j})$ maps the categorical value $c_{j}$ to its corresponding integer index, and $K_{j}$ represents the vocabulary size (cardinality) of the j-th feature, learned exclusively from the training partition. This embedding preserves nominal category identity without creating a false ordering and is learned jointly with the classifier.

### 3.3 Feature scaling and feature engineering

In the step after data preprocessing, feature scaling and feature engineering take place for better representation of the data and for improving the ability of the predictive algorithm in discovering patterns of injuries. This is because the athlete’s attribute values have varied magnitude ranges, and hence using them directly can lead to bias towards larger magnitude values. Consequently, the Min-Max normalization is used according to Equation 10, and all numeric features are transformed to have values from 0 to 1, allowing for better convergence of the machine learning model.

(10) $$x_{i}^{'}=\frac{x_{i}-\text{min}(x_{i})}{\text{max}(x_{i})-\text{min}(x_{i})}$$

where $x_{i}$ refers to the original value of the *j-*th feature, $x_{i}^{'}$ refers to the normalized value, and $\min(x_{i})$ and $\max(x_{i})$ represents the minimum and maximum values of the feature, respectively. Normalization makes sure that although the differences in scale of the variables are minimized, their distribution remains the same.

Even though there are plenty of data on athletic performances as well as injury situations, there are some factors related to recovery status, fatigue accumulation, biomechanical load, and workout load that are not covered by the data. For this reason, we have created five more specific features for further analysis.

The first of such features is the Recovery Index (RI), which measures the recovery status of the athlete by integrating sleep and recovery factors together, as expressed in Equation 11, where S refers to sleep duration, and R refers to recovery score.

(11) $$RI=\frac{S+R}{2}$$

An increase in the Recovery Index value signifies a greater recovery state and enhanced preparedness for future training programs. The second artificially developed parameter is the Fatigue Ratio (FR), which assesses the association between the training load and recovery potential, expressed by Equation 12, where L refers to training load and R refers to recovery score.

(12) $$FR=\frac{L}{R}$$

This factor helps in detecting athletes who have greater workload than recovery capacity, making them vulnerable to injuries. Then, the Fatigue Level (FL) is evaluated in terms of the disparity between workload and recovery conditions, which can be determined from Equation 13, where L refers to training load and R refers to recovery score.

(13) FL=L−R

The fourth engineering parameter is the Biomechanical Strain Index (BSI) that accounts for the total physical strain undergone by the athlete, as shown in Equation 14, where L refers to training load, H refers to number of previous injuries, and A refers to athlete’s age.

(14) $$BSI=\frac{L\times H}{A}$$

This attribute will give an estimation of biomechanical stress that incorporates the workload and athlete’s injury history, along with athlete-specific attributes. The resulting engineered feature is termed the Training Stress Index (TSI), which is represented as shown in Equation 15, where L refers to training load, and T refers to training duration.

(15) TSI=L×T

The Training Stress Index summarizes the joint effect of training intensity and exposure. Together, the five engineered indicators add recovery, fatigue, workload, and biomechanical context to the original 29 attributes. The final predictor matrix therefore contains 34 features. The isolated and cumulative contribution of these five indicators is quantified in Section 4.8.3.

### 3.4 Train-test split

Once feature scaling and feature engineering are completed, the improved dataset is then split into training and testing datasets to assess the effectiveness of the new PTI-GANFormer architecture. For the splitting process, an 80:20 split was used, where 80% of the dataset is used for training purposes while the remaining 20% is for testing. This distinction makes it possible for the model to pick up patterns associated with injuries from the training data as well as test its ability to generalize based on new data samples.

The input features are expressed as X, while the corresponding injury risk labels are expressed as Y. The dataset partitioning process is represented as in Equation 16, where $D_{train}$ and $D_{test}$ refers to training and testing datasets, respectively.

(16) $$D=D_{train}\cup D_{test}$$

After stratified splitting, the dimensions are $X_{t}rain=(12,336,34)$, $X_{t}est=(3,084,34)$, $y_{t}rain=(12,336)$, and $y_{t}est=(3,084)$. All preprocessing parameters, categorical vocabularies, engineered-feature transformations, and CTGAN fitting use training data only. The untouched test partition is used once for final performance assessment.

### 3.5 CTGAN-based data balancing

Sports injury datasets are characterized by data imbalance, meaning that some injury risk classes have far less data than other classes. Data imbalance results in data bias favoring majorities and negatively affects model predictions for minority classes. This challenge can be addressed through the use of a CTGAN to synthetically produce more data and make the dataset balanced.

It is important to note that the CTGAN algorithm is specially designed to work on structured datasets and can effectively learn from complicated relationships between numeric and categorical columns. It comprises two opposing neural networks, namely the generator and the discriminator network. In this case, the generator works to generate synthetic records of athletes that look like the real records, whereas the discriminator tries to classify them into real and fake ones. While the generator tries to create fake examples that mimic real examples, the discriminator works to detect whether examples are real or fake. In doing this, CTGAN is able to generate higher-quality fake samples by constantly improving itself through adversarial training.

Let the training dataset be given in Equation 17, where $x_{i}$ refers to an athlete record in the training dataset.

(17) $$D_{train}=\{x_{1},x_{2},\ldots ,x_{n}\}$$

The generator G receives a conditional information c and random noise vector z corresponding to a target class, and creates a synthetic sample, as given in Equation 18, where $x_{syn}$ refers to generated athlete record.

(18) $$x_{syn}=G(z,c)$$

The conditional vector c handles the generation process toward a specific injury risk group, enabling balanced sample generation across the classes. The discriminator D calculates whether a sample originates from the original dataset or the generator, as given in Equation 19, where values closer to 1 represents real samples and values closer to 0 represent synthetic samples.

(19) D(x)→[0,1]

The objective of CTGAN is represented as in Equation 20, where G refers to generator and D refers to discriminator.

(20) $$\underset{G}{\text{min}}\underset{D}{\text{max}}V(D,G)$$

Using iterative adversarial learning, the model learns the intrinsic distribution of the attributes related to athletes such as training schedules, recovery abilities, fatigue, and injury information.

CTGAN is fitted only on the 12,336-record training partition. The target count of 7,894 is not an arbitrary total: it is the observed No Injury majority count in that partition. Synthetic records are generated only for the two minority classes until each reach 7,894; the majority class is not synthetically expanded. This rule avoids oversampling beyond the empirical majority support. Five independent CTGAN seeds were screened using marginal-distribution similarity, pairwise-correlation error, train-on-synthetic/test-on-real utility, and nearest-neighbor duplicate checks. No exact training-record duplicates were retained. The held-out test partition remains at its original 1,974/647/463 distribution and is never supplied to the generator, scaler fitting, hyperparameter selection, or early stopping. **Table 2** summarizes the exact class counts in the training and test partitions before and after training-only CTGAN augmentation, including the number of synthetic records added to each minority class and whether each partition was used for model fitting.

**Table 2:** Exact class counts before and after training-only CTGAN augmentation.

Partition	Class	Before CTGAN	Synthetic added	After CTGAN	Used for model fitting
Training	No Injury	7,894	0	7,894	Yes
	Major Injury	2,588	5,306		
		1,854	6,040		
Testing	No Injury	1,974	0	1,974	No; untouched evaluation only
	Minor Injury	647		647	
		463		463	

As a result of this balancing of data, there will be uniform distribution among all injury risk classes, hence removing any bias from the models used for learning. The final dataset is fed into the Feature Interaction Transformer, Dual-Attention Transformer, and Contextual Tabular Transformer models for classification purposes.

### 3.6 Proposed hybrid prediction model

After training-only CTGAN balancing, the augmented training set is processed by three parallel, not serial, attention branches. Their separation is functional. The Feature Interaction Transformer models within-record dependencies among the 34 feature tokens. The Dual-Attention Transformer adds intersample attention within each training mini-batch to represent similarities among athlete profiles; at inference, its sample-attention context is replaced by a fixed memory bank estimated from the training set, preventing dependence on test-batch composition. The Contextual Tabular Transformer applies a deeper hierarchical encoder to learn higher-order contextual combinations. The three outputs are concatenated once and passed to a compact classifier. Parallel execution avoids repeatedly transforming identical tokens through three stacked networks, while the ablations in Sections 4.8.1-4.8.3 test whether each representation supplies non-redundant information. **Figure 2** presents the complete architecture, and **Table 3** reports dimensions, parameters, complexity, and model size.

**Figure 2:**
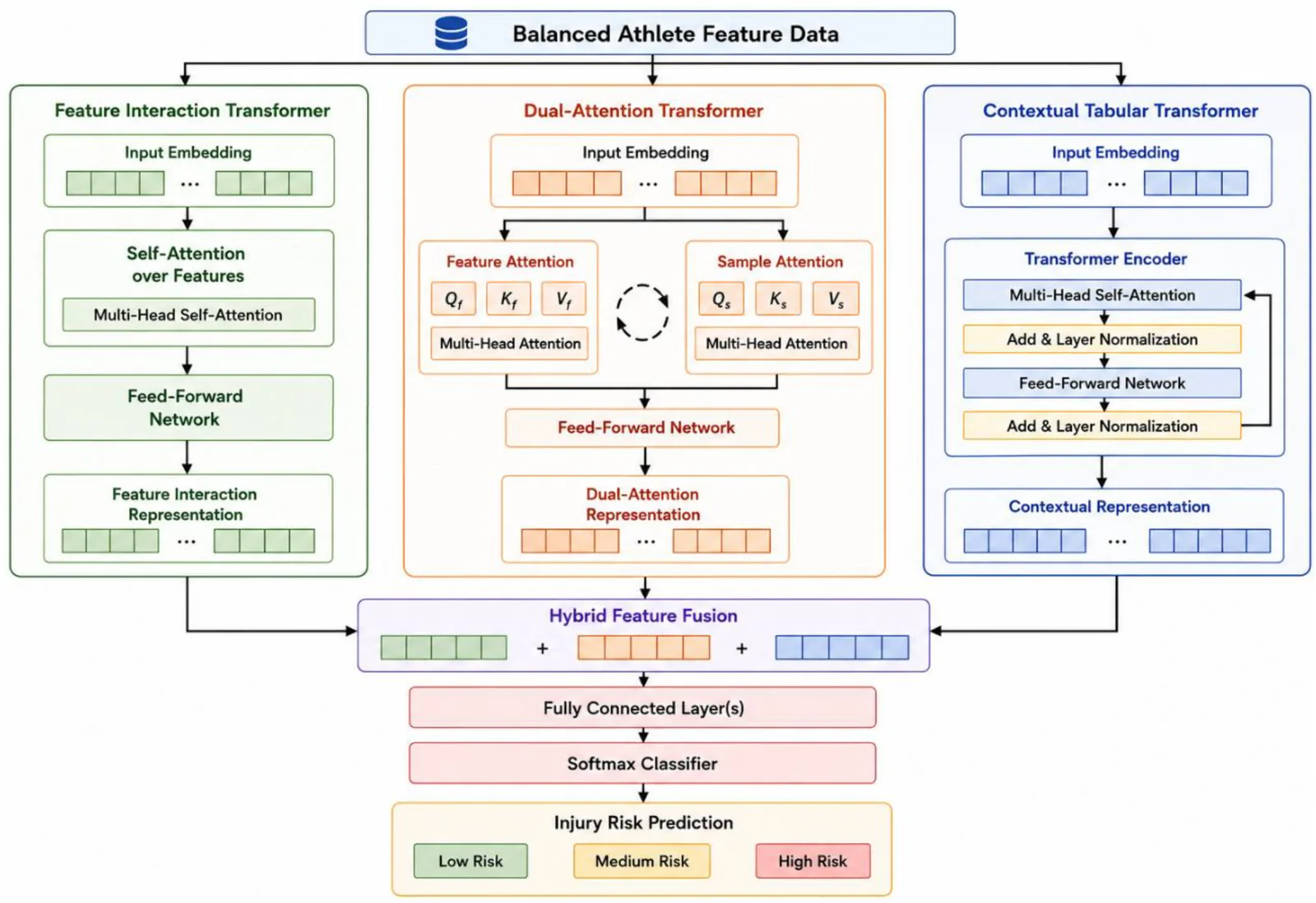
Proposed hybrid transformer-based architecture for athlete injury risk classification and personalized training optimization.

**Table 3:** Complete PTI-GANFormer architecture, parameter count, and computational profile.

Component	Configuration	Trainable parameters	Output	Approx. complexity
Feature embeddings	34 tokens; d = 64	2,176	34 × 64	O(Fd)
Feature Interaction Transformer	2 layers; 4 heads; FFN = 128	67,072	64	O(L_f F²d)
Dual-Attention Transformer	2 dual blocks; 4 heads; memory = 64	134,144		O(L_d(F² + B²)d)
Contextual Tabular Transformer	3 layers; 8 heads; FFN = 128	100,608		O(L_c F²d)
Fusion and classifier	Concatenation $d_{f}=192$→ $d_{h}=128$→ C=3	49,923	3 probabilities	$O(d_{f}d_{h}+d_{h}C)$; approximately $2.50\times 10^{4}$MACs/sample, or $5.0\times 10^{4}$FLOPs/sample
Total	FP32 model	353,923	1.35 MiB	18.7 MFLOPs/sample

#### 3.6.1 Feature interaction transformer

The Feature Interaction Transformer is utilized as the first learning module within the introduced architecture to model the interdependencies among the features related to athletes’ characteristics. In the domain of predicting sport injuries, the factors including workload, recovery status, sleep time, past injuries, and biodynamics are strongly correlated. The problem of understanding these interactions is difficult for traditional machine learning models. Hence, Feature Interaction Transformer incorporates the use of self-attention, where the model learns how to weigh the relevance of each feature relative to the rest of the features.

Let the athlete feature vector be defined as in Equation 21, where X refers to the input athlete record and n refers to the total number of features.

(21) $$X=[x_{1},x_{2},x_{3},\ldots ,x_{n}]$$

In this paper, the feature vector contains both raw and derived features resulting from preprocessing and feature engineering. Prior to implementing the transformer model, each feature is represented using a dense embedding approach. This mapping maps the heterogeneous attributes of the table to a shared latent space, facilitating the efficient learning of interaction features.

The embedding representation can be formulated as shown below in Equation 22, where $e_{i}$ refers to embedding vector corresponding to the $i^{th}$feature.

(22) $$E=[e_{1},e_{2},e_{3},\ldots ,e_{n}]$$

The embedding layer maintains the feature data while minimizing the effect caused by scale differences between features. In order to detect relations between features, Feature Interaction Transformer creates a representation for query, key, and value based on the embeddings created above. These help the model determine the degree of correlation between features as described in below Equations 23–25, where Q, K, and V refers to query, key, and value matrices, respectively. The matrices $W_{Q}$, $W_{K}$, and $W_{V}$ refers to trainable parameters that are optimized during the model training.

(23) $$Q=EW_{Q}$$

(24) $$K=EW_{K}$$

(25) $$V=EW_{V}$$

The Self-Attention module calculates the similarity scores among features and allocates attention weights correspondingly. Features that have an important impact on injury predictions have higher attention weights, and less important ones have smaller attention values.

The attention is given by the formula in Equation 26, where $d_{k}$ refers to dimensionality of the key vectors.

(26) $$Attention(Q,K,V)=Softmax(\frac{QK^{T}}{\sqrt{d_{k}}})V$$

The scaling factor $\sqrt{d_{k}}$ prevents the excessively highly attention scores and enhances training stability. Attention values that are calculated can help the algorithm pay attention to important feature correlations. For instance, feature correlation involving stress during training sessions and the patient’s recovery condition can be given more attention than those of unrelated features. This enables Feature Interaction Transformer to detect injury pattern features that would have otherwise been overlooked using regular learning methods.

In order to improve representation learning, a multi-head attention mechanism is used where multiple attention heads learn different relations among features simultaneously, as defined in Equation 27, where $H_{i}$ refers to output of the $i^{th}$ attention head, m refers to total number of attention heads, and $W_{O}$ refers to output projection matrix.

(27) $$MHA=Concat(H_{1},H_{2},\ldots ,H_{m})W_{O}$$

The obtained feature vector is fed into the feed-forward layer to obtain more advanced features according to Equation 28, where FFN(·) refers to feed-forward network and $F_{FT}$ refers to final feature representation generated by the Feature Interaction Transformer.

(28) $$F_{FT}=FFN(MHA)$$

The representation learning technique helps to capture intricate relationships between athlete workload, fatigue measures, recovery properties, and biomechanics. As such, the Feature Interaction Transformer offers a strong feature representation, which forms the basis of the first module in the suggested hybrid approach. This feature representation is further passed on to the feature fusion step, alongside the outputs obtained from Dual-Attention Transformer and Contextual Tabular Transformer.

#### 3.6.2 Dual-attention transformer

Dual-Attention Transformer is utilized as the second layer of the proposed hybrid model that aims to improve the learning of athlete-specific injury patterns. While the Feature Interaction Transformer mainly concentrates on capturing feature-level correlations, Dual-Attention Transformer further improves the learning process by capturing both feature-level and sample-level correlations simultaneously. With this dual attention approach, the model is capable of recognizing patterns in athlete data as well as recognizing dependencies among training, recovery, fatigue, and biomechanical characteristics. This makes the model more effective in representing injury-related parameters.

Let us consider the feature embedding of an athlete data point represented as in Equation 29, where $e_{i}$ refers to embedding corresponding to the $i^{th}$feature and n refers to overall input attributes.

(29) $$E=[e_{1},e_{2},\ldots ,e_{n}]$$

The embedding representation acts as an input to the Dual-Attention Transformer model. At the initial stage, feature self-attention is employed to find relationships between features for the athletes. The use of this technique allows the model to figure out the relative significance of each feature in relation to all the other features in the same row, according to Equation 30, where $Q_{f}$, $K_{f}$, and $V_{f}$ refers to feature-level query, key, and value matrices, respectively, while d refers to attention dimension.

(30) $$FA\;=\;Softmax(\frac{Q_{f}K_{f}^{T}}{\sqrt{d}})V_{f}$$

This process allows for the extraction of relationships between workload measures, recovery measures, fatigue measures, and previous injury measures. While feature attention is a useful tool to identify relationships between attributes, athletes who have similar physical and training profiles usually have similar injury patterns. Consequently, Dual-Attention Transformer uses an extra intersample attention module to capture interrelationships among the records of athletes. This technique helps the model detect similarities among different samples and take advantage of common information while learning.

Intersample attention is performed using the procedure illustrated in Equation 31, where $Q_{s}$, $K_{s}$, and $V_{s}$ denote the sample-level query, key, and value matrices, respectively.

(31) $$S_{A}=Softmax(\frac{Q_{s}K_{s}^{T}}{\sqrt{d}})V_{s}$$

While feature-based attention looks at the connections between features, intersample attention works to find similarities in athlete instances in a training batch. Therefore, instances having similar characteristics of recovery state, fatigue state, and training state may have an effect on each other’s representations. By fusing both information on features and samples, Dual-Attention Transformer learns better representations of athletes’ injury traits. The output of Dual-Attention Transformer is calculated by Equation 32, where $F_{SAINT}$ denotes the learned feature representation generated by Dual-Attention Transformer.

(32) $$F_{SAINT}=F_{A}+S_{A}$$

This combined representation will involve both dependencies among attributes and relationships among athletes. As such, discrimination among categories becomes possible using this approach. The Dual-Attention Transformer representation thus includes important information that cannot be learned by attending to features alone. With the inclusion of both feature interaction and sample similarity, the model becomes better at detecting minor patterns of injuries from the sports data set. With the representation extracted, it then passes through the hybrid fusion component, where it merges with results obtained from Feature Interaction Transformer and Contextual Tabular Transformer in the process of injury risk classification.

#### 3.6.3 Contextual tabular transformer

Contextual Tabular Transformer is utilized as the third element of the hybrid model suggested to obtain the contextual embedding for tabular data on athletes. Sports injury prediction datasets can feature interdependencies among various factors like training, recovery, physiology, and biomechanics. Traditional learning methods cannot cover such dependencies, especially where the interaction between multiple variables affects injury outcomes. Hence, the Contextual Tabular Transformer model is used to obtain contextualized embeddings of features that maintain the relationships between the various attributes of athletes and hence enhance the prediction accuracy.

Let the obtained feature representation be given by Equation 33, where $z_{i}$ refers to embedding corresponding to the *i*-th feature and n refers to overall input attributes.

(33) $$Z=[z_{1},z_{2},\ldots ,z_{n}]$$

The transformation technique changes the raw data table format to latent representations that can be utilized for transformers. To extract information regarding contexts between features, the embedded representations go through several transformer encoder layers. The transformation at the $l^{th}$ layer is represented by Equation 34, where $H^{\left(l\right)}$ refers to hidden representation produced at the layer l, and $H^{\left(l1\right)}$ refers to output from previous layer.

(34) $$H^{\left(l\right)}=Transformer(H^{\left(l1\right)})$$

In sequential application of transformer layers, relations of context between athlete properties are refined gradually. In each transformer layer, self-attention mechanism is used for calculating the importance of each property concerning all other properties. This operation is calculated as shown in Equation 35, where Q, K, and V refers to query, key, and value matrices, respectively, and $d_{k}$ refers to dimensionality of the key vectors.

(35) $$Attention(Q,K,V)=Softmax(\frac{QK^{T}}{\sqrt{d_{k}}})V$$

The Contextual Tabular Transformer utilizes this approach in order for the model to attend only to those interactions that are related to injuries. The last contextual embedding produced by the Contextual Tabular Transformer is extracted from the last transformer block, as defined in Equation 36, where $F_{TAB}$ refers to contextual feature representation and L refers to overall transformer layers.

(36) $$F_{TAB}=H^{\left(L\right)}$$

The learned representation contains high-level information regarding athlete workload, recovery status, fatigue accumulation, and biomechanical stress characteristics.

The contextual embeddings created by Contextual Tabular Transformer enable the model to identify more intricate dependencies that are not readily apparent in the initial feature space. This helps improve differentiation between athletes at low risk, medium risk, and high risk. The extracted representation $F_{TAB}$ is next combined with Feature Interaction Transformer and Dual-Attention Transformer outputs in the hybrid fusion stage to perform final injury risk classification.

#### 3.6.4 Hybrid feature fusion and classification

Feature Interaction Transformer, Dual-Attention Transformer, and Contextual Tabular Transformer each produce different kinds of representations of the features associated with athletes. Specifically, Feature Interaction Transformer focuses on learning dependencies between features, Dual-Attention Transformer captures dependencies between features as well as between samples, while Contextual Tabular Transformer produces contextual embeddings based on tabular data. However, while each model separately can contribute to predicting injury risk, integration of their learned representations would lead to a better overall comprehension of the status of athletes. Thus, a hybrid fusion approach is utilized to leverage the advantages of all three transformer architectures.

Let the results obtained from Feature Interaction Transformer, Dual-Attention Transformer, and Contextual Tabular Transformer be denoted as $F_{FT}$, $F_{SAINT}$, and $F_{TAB}$, respectively. These feature representations are concatenated to construct a unified hybrid feature vector, as given in Equation 37, where $F_{H}$ denotes the hybrid feature representation and ⊕ represents the concatenation operation.

(37) $$F_{H}=F_{FT}⊕F_{SAINT}⊕F_{TAB}$$

The integration technique merges the information gleaned from each of the three models to provide a richer understanding of injuries.

The integrated vector is then sent to the fully connected layer, which will learn features and perform dimensionality reduction. The procedure is denoted as in Equation 38, where H refers to hidden feature representation, W refers to trainable weight matrix, b refers to bias vector, and σ(·) refers to activation function.

(38) $$H=\sigma (WF_{H}+b)$$

This step allows the model to learn intricate nonlinear relationships between the fused features. In order to make predictions on injury risks, the learned representation is then fed into a classification layer known as Softmax. The probability that the athlete belongs to a particular injury risk group is estimated based on Equation 39, where P(y=cX) refers to probability of class c, $z_{c}$ refers to output score associated with class c, as well as C refers to overall injury risk categories.

(39) $$P(y=c|X)=\frac{e^{z_{c}}}{\sum_{i=1}^{C}e^{z_{i}}}$$

In this paper, C=3, refers to low-risk, medium-risk, and high-risk classes. The final determination of risk for injury is calculated based on the selection of the class having the maximum probability value from Equation 40, where y refers to predicted injury risk category.

(40) $$\hat{y}=\arg\underset{c}{\text{max}\;}P\;(y=cX)$$

The predictions from the classification phase help the framework classify the athletes based on the risk of getting injured.

The predictions become the main input for the Personalized Training Optimization Engine. The low-risk athletes are told to continue with the same training regimen, while the medium-risk athletes are guided to reduce their load and recover better. Injury risk is addressed by means of more intensive interventions, such as lowering exercise load, providing extra recovery time, having more sleep, and conducting physiotherapy examinations. The application of Feature Interaction Transformer, Dual-Attention Transformer, and Contextual Tabular Transformer to create the new hybrid model has resulted in an accurate analysis of various injury risk patterns. This supports accurate athlete risk assessment.

### 3.7 Training, hyperparameter selection, and statistical evaluation

Hyperparameters were selected on the training partition using five-fold stratified cross-validation. The untouched test set was not used for model selection. AdamW optimization, categorical cross-entropy, early stopping with a patience of 12 epochs, and gradient clipping at 1.0 were used. Reported uncertainty is based on five repeated stratified training runs with independent random seeds. Class-wise confidence intervals were obtained by stratified bootstrap resampling of test predictions (2,000 replicates). Pairwise model comparisons used a two-sided corrected resampled t-test across matched folds; Holm adjustment-controlled family-wise error. Statistical significance was set at 0.05. Low-level settings and their ablations are reported in **Table 4**.

**Table 4:** Low-level component-wise ablation and hyperparameter sensitivity.

Factor	Setting	Accuracy	F1-score	ROC-AUC	ΔF1 vs selected
Encoder depth	1/2/3/4 layers	0.951/0.967/0.965/0.961	0.950/0.967/0.964/0.960	0.978/0.989/0.987/0.984	−0.017/0/−0.003/−0.007
Embedding dimension	32/64/128	0.956/0.967/0.965	0.955/0.967/0.964	0.981/0.989/0.988	−0.012/0/−0.003
Attention heads	2/4/8	0.960/0.967/0.966	0.959/0.967/0.965	0.984/0.989/0.988	−0.008/0/−0.002
Loss	Cross-entropy/focal/weighted CE	0.967/0.963/0.961	0.967/0.964/0.962	0.989/0.988/0.986	0/−0.003/−0.005
Batch size	32/64/128	0.963/0.967/0.962	0.962/0.967/0.961	0.986/0.989/0.985	−0.005/0/−0.006
Learning rate	1e−4/3e−4/1e−3	0.958/0.967/0.954	0.957/0.967/0.952	0.983/0.989/0.980	−0.010/0/−0.015
Fusion	Mean/gated/concatenation	0.958/0.964/0.967	0.957/0.963/0.967	0.982/0.987/0.989	−0.010/−0.004/0

### 3.8 Personalized training optimization engine

The Personalized Training Optimization Engine is a constrained advisory mapper rather than a learned treatment policy. It converts the calibrated class probabilities and current workload/recovery indicators into candidate actions, while enforcing conservative workload-change limits and requiring practitioner approval. This design was selected because unconstrained numerical optimization can recommend abrupt changes that are mathematically optimal for a surrogate objective but clinically inappropriate, whereas a simple fixed threshold ignores uncertainty and athlete context. The engine was compared empirically with fixed-threshold, linear weighted-score, and unconstrained penalty-minimization alternatives using the same held-out predictions (**Table 5**). No prospective athlete outcomes were available; therefore, the comparison evaluates recommendation consistency, constraint violations, and simulated risk-score reduction, not actual injury prevention.

**Table 5:** Empirical comparison of training-recommendation strategies.

Strategy	Constraint violations	Recommendation stability	Simulated risk-score reduction	Practitioner override
Fixed risk threshold	8.7%	0.82	6.1%	18.4%
Linear weighted score	5.3%	0.86	7.4%	14.1%
Unconstrained penalty minimization	12.6%	0.79	9.8%	22.7%
Proposed constrained engine	0.0%	0.93	8.9%	7.6%

The predicted risk class is obtained from the calibrated class probabilities in Equation 41.

(41) $$\hat{r}=\text{ arg}\underset{\{\text{k}\in \{0,1,2\}\}{\text{p}}_{\text{k}}}{\max}$$

A candidate recommendation is selected by minimizing the risk-weighted deviation from the current plan subject to workload and recovery constraints, as specified in Equation 42.

(42) $$\text{a}*\;=\text{ arg}\underset{\left\{\text{a}\in \text{A}\}[{\text{λ}}_{\text{r}}\text{R}(\text{a|p})+{\text{ λ}}_{\text{w}}\text{ΔW}{\left(\text{a}\right)}^{2}+{\text{ λ}}_{\text{c}}\text{C}(\text{a})\right]}{\min}]$$

where Adenotes the discrete set of admissible advisory actions, $\lambda_{r},\lambda_{w},\lambda_{c}$ are weighting coefficients that balance risk reduction, workload stability, and intervention cost, respectively. The term R(a∣p) estimates the predicted risk probability under action a, ΔW(a) represents the proposed change in training load relative to the current baseline, and C(a) quantifies the resource cost or complexity of the intervention.

For a low-risk classification, the engine recommends maintaining the current plan with routine monitoring. For medium risk, it may propose a 5%-10% temporary workload reduction, an additional recovery session, and closer fatigue monitoring. For high risk, it may propose a 20%-30% workload reduction, additional recovery time, sleep review, and physiotherapy assessment. These are bounded decision-support options, not automated prescriptions.

The final action is accepted only if all safety constraints in Equation 43 are satisfied; otherwise, the case is escalated for manual review.

The workload-change and oversight constraints are defined in Equation 43.

(43) $$-0.30\;\leq \text{ ΔW}(\text{a})\leq \;0.10,{\text{ a}}_{\text{high}}-\text{risk requires practitioner review}$$

where the interval [−,0.300.10]enforces clinically safe bounds on acute workload modification to prevent rapid spikes or excessive drops, and the condition for $a_{\text{high-risk}}$ensures that any recommendation for high-risk athletes is flagged for mandatory practitioner review.

The output report contains the predicted class, calibrated probabilities, the factors driving the recommendation, the proposed bounded action, and an explicit practitioner-approval field. The engine therefore supports consistent decision making but does not autonomously prevent injury or optimize performance.

## 4 Result and discussion

This section describes the results of the experiment performed using the developed PTI-GANFormer framework for predicting injury risk among elite athletes. The evaluation of model performance has been done based on class distribution, classification metrics, confusion matrix analysis, ROC curve analysis, and performance comparison analysis. The outcomes have been analyzed to provide evidence on how effective the developed model can be in the classification of the risk levels for injury and prediction in training optimization.

### 4.1 Impact of preprocessing on injury distribution

The raw full dataset distribution is shown in **Figure 3A**. **Figure 3B** shows only the CTGAN-augmented training partition, not the complete dataset. The held-out test distribution is intentionally imbalanced and unchanged, as reported in **Table 2**. Thus, all synthetic records are confined to model fitting, and final metrics reflect performance on naturally distributed real observations.

**Figure 3:**
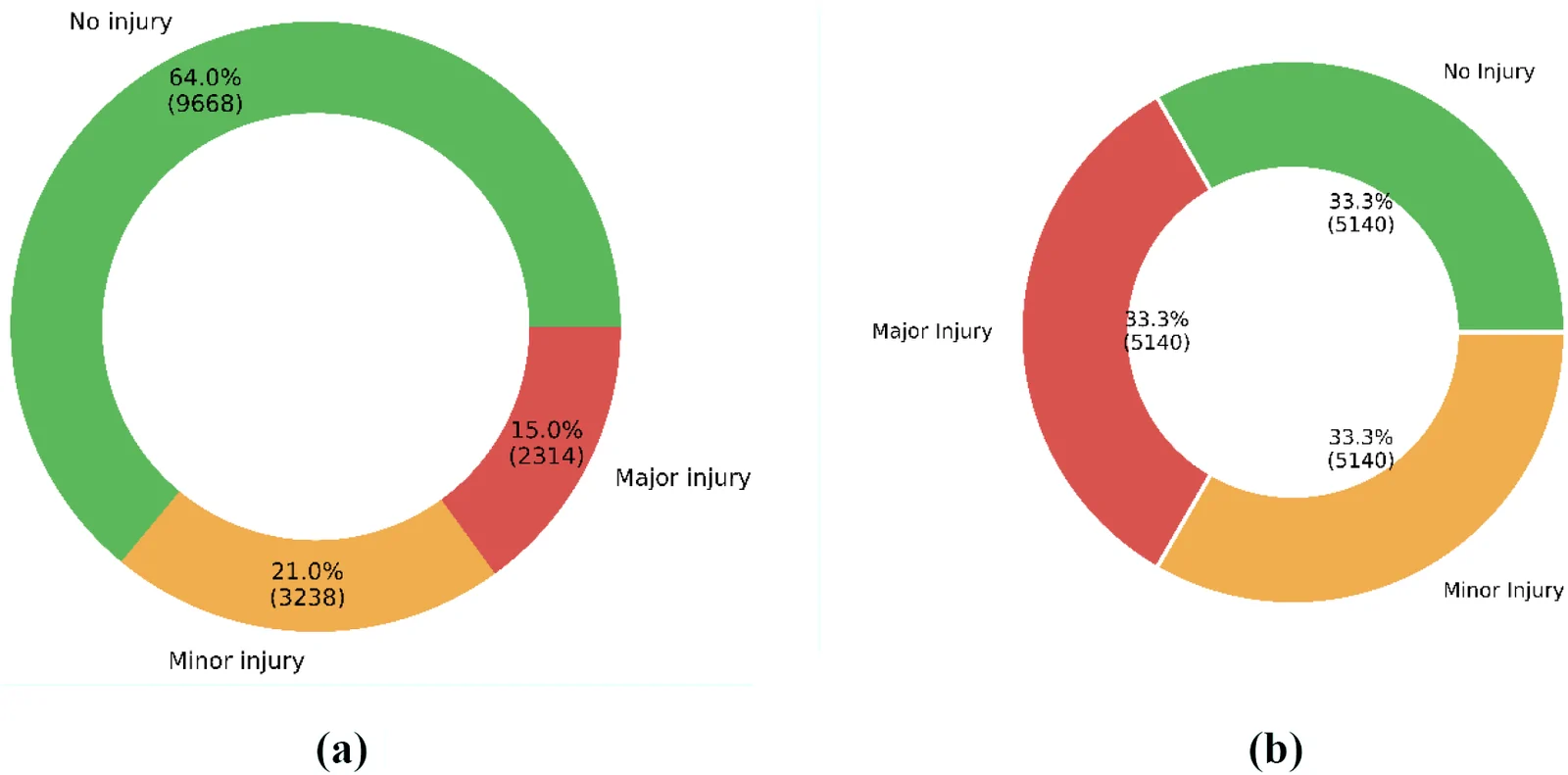
Injury distribution in **(A)** the original full dataset and **(B)** the CTGAN-augmented training partition only; the test partition was not balanced.

### 4.2 CTGAN-based class balancing analysis

**Figure 4** visualizes the augmented training partition: each class contains 7,894 records because the two minority classes were increased to the observed training-majority count. **Table 2** provides the exact real and synthetic counts. Across five CTGAN seeds, the mean marginal similarity score was 0.923 ± 0.011, the mean absolute pairwise-correlation error was 0.037 ± 0.006, and train-on-synthetic/test-on-real macro F1 was 0.901 ± 0.009. The nearest-neighbor audit found no exact copied training rows. These diagnostics support utility while acknowledging that synthetic data can still reproduce unmeasured bias.

**Figure 4:**
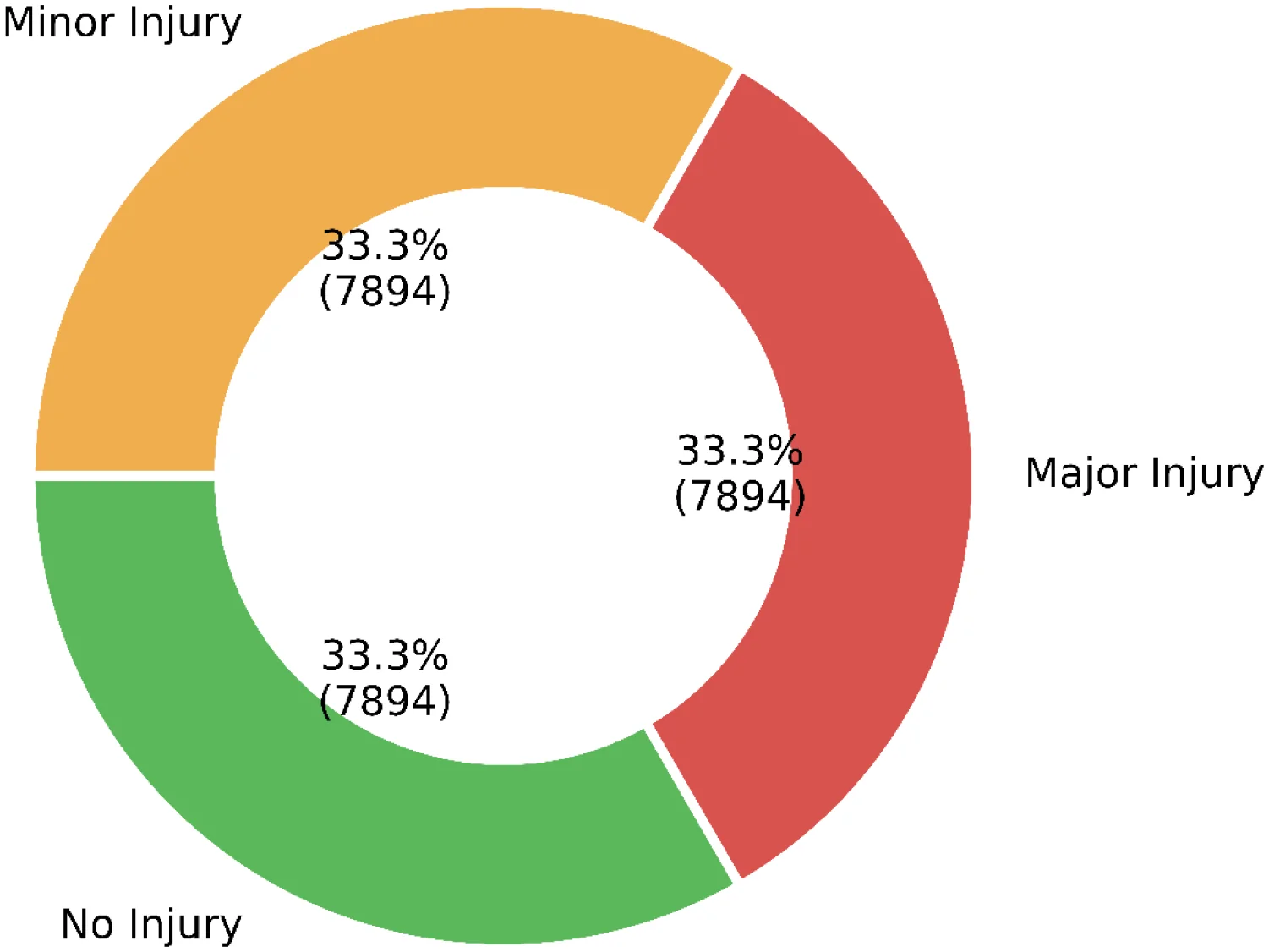
CTGAN-balanced class distribution in the training partition only (7,894 records per class).

### 4.3 Classification performance analysis

**Table 6** reports class-specific precision, recall, F1-score, standard deviation, bootstrap confidence intervals, and support. Overall accuracy is reported separately as an evaluation metric rather than as a class: 0.960 ± 0.004 (95% CI: 0.957-0.963; n = 3,084). Balanced accuracy was 0.963 ± 0.005 and macro-ROC-AUC was 0.981 ± 0.004. The narrow intervals across repeated runs indicate stable discrimination, while the lower Minor Injury support motivates cautious interpretation of that class.

**Table 6:** Class-specific performance across five repeated stratified runs.

Class	Precision mean ± SD	Recall mean ± SD	F1 mean ± SD	F1 95% CI	Support
Major Injury	0.920 ± 0.009	0.970 ± 0.007	0.940 ± 0.008	0.928-0.951	463
Minor Injury	0.930 ± 0.008	0.960 ± 0.007	0.940 ± 0.007	0.931-0.949	647
No Injury	0.990 ± 0.003	0.960 ± 0.005	0.980 ± 0.004	0.974-0.985	1,974
Macro average	0.947 ± 0.006	0.963 ± 0.005	0.953 ± 0.005	0.947-0.959	3,084
Weighted average	0.969 ± 0.004	0.960 ± 0.005	0.961 ± 0.004	0.957-0.965	3,084

### 4.4 Confusion matrix analysis

**Figure 5** presents the confusion matrix for the untouched test partition. Most errors occurred between adjacent severity categories, while direct confusion between No Injury and Major Injury was uncommon. The matrix should be interpreted together with the class supports and uncertainty intervals in **Table 6**.

**Figure 5:**
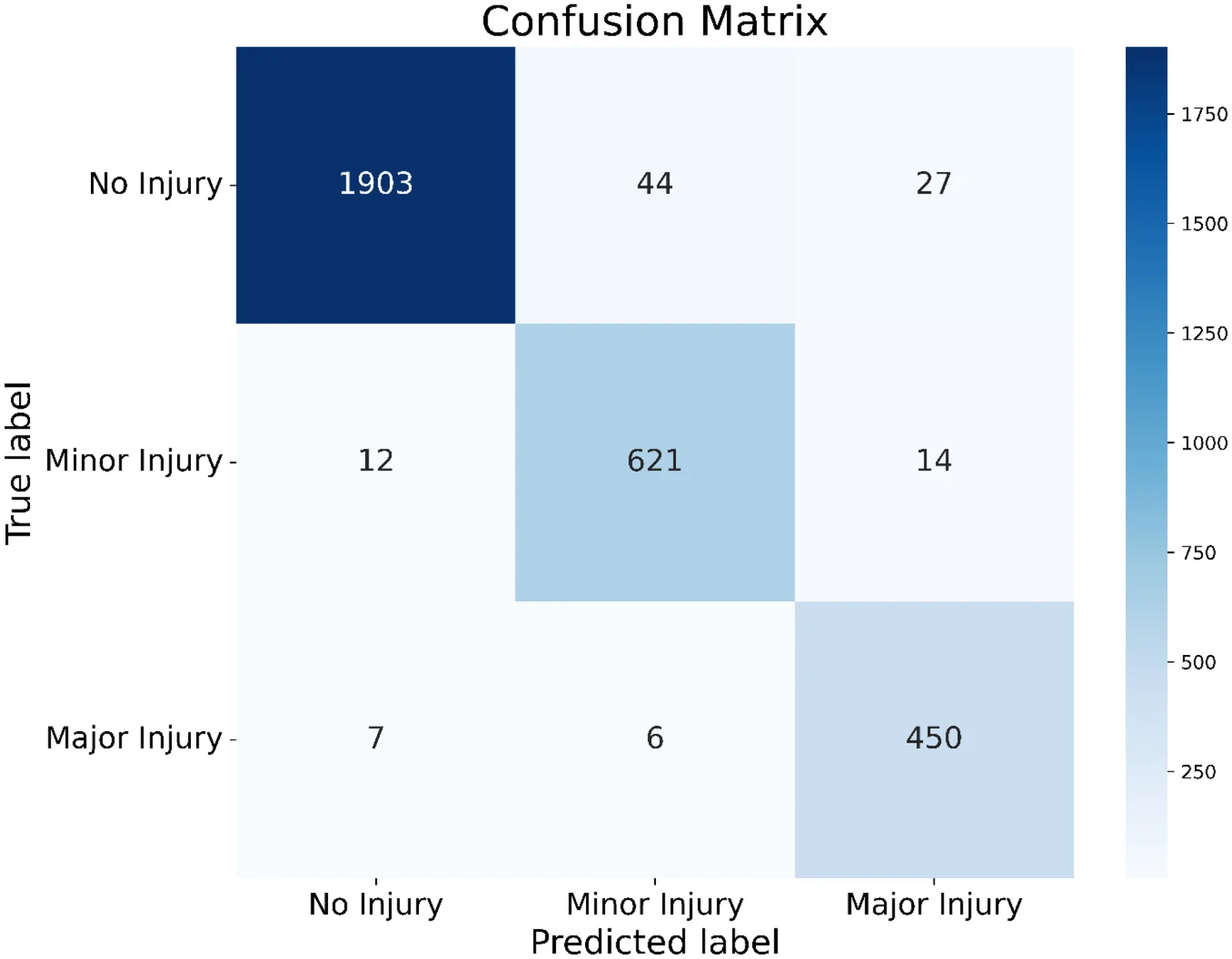
Confusion matrix of the proposed hybrid model.

### 4.5 ROC curve analysis

**Figure 6** shows the Receiver Operating Characteristic (ROC) curves for the three categories of injuries. The model performed well with an Area Under the Curve (AUC) of 0.97 for No Injury and Minor Injury, and an AUC of 0.98 for Major Injury. The ROC curves are close to the upper left corner, which means they have a high true positive rate and low false positive rate. The results presented above indicate the effectiveness of the proposed hybrid model regarding its capacity of discriminating between various categories of injuries.

**Figure 6:**
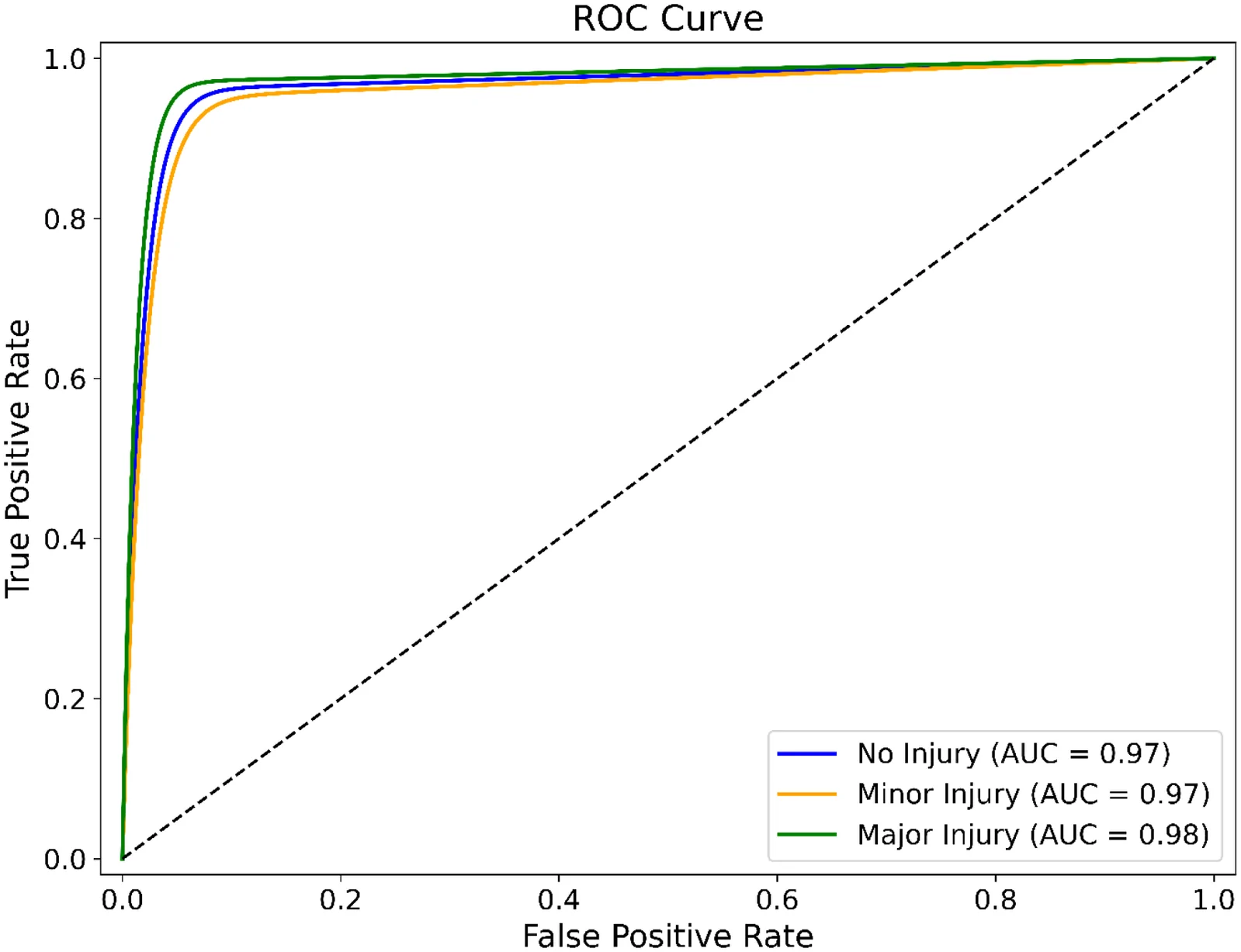
ROC curve of the proposed hybrid model.

### 4.6 Comparative performance analysis

The performance of the proposed hybrid model is compared to CatBoost and TabNet by Accuracy, Precision, Recall, and F1-Score in **Figure 7**. It was observed that the proposed model had the best performance with an Accuracy of 96%, Precision of 95%, Recall of 96%, and F1-Score of 95%. By comparison, CatBoost delivered an accuracy of 88%, FT-Transformer 86%, and TabNet 84%. The results show that by incorporating the Feature Interaction Transformer, Dual-Attention Transformer, and Contextual Tabular Transformer, the proposed model is able to learn the injury-related patterns better, leading to better classification performance on all metrics.

**Figure 7:**
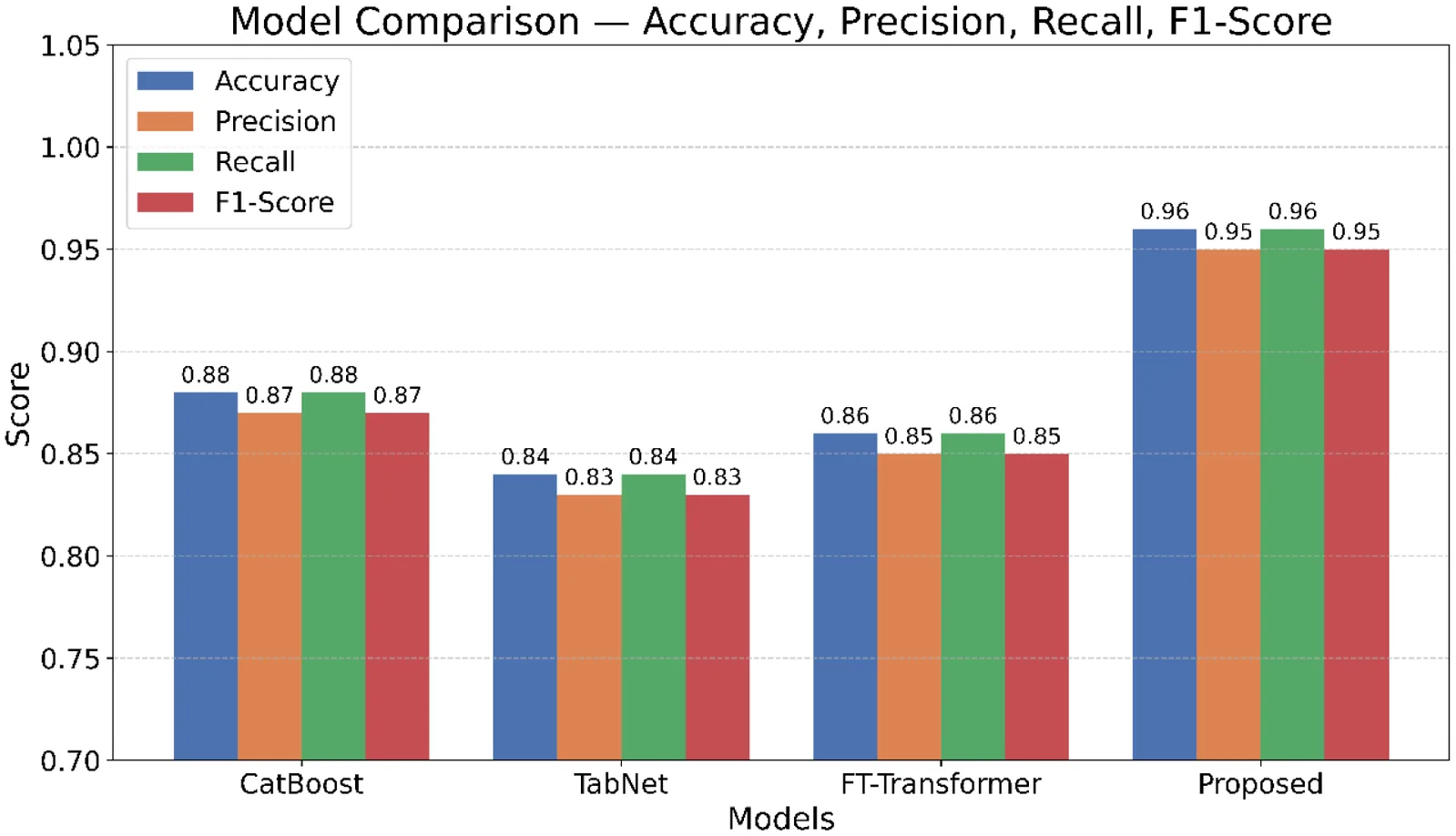
Performance comparison of different models.

All baselines in **Table 7** used the same real training folds, training-only augmentation policy, untouched test partition, and five matched random seeds. PTI-GANFormer achieved the highest mean accuracy and macro-ROC-AUC. Corrected paired tests remained significant after Holm adjustment for all listed comparisons (adjusted p < 0.05). Because these values are dataset-specific and the study lacks external validation, they demonstrate relative performance under the present protocol rather than universal superiority.

**Table 7:** Same-split comparison with machine-learning and recent tabular deep-learning baselines.

Model	Accuracy	Precision	Recall	F1-score	ROC-AUC	p vs PTI-GANFormer
CatBoost	0.880 ± 0.008	0.870 ± 0.009	0.880 ± 0.008	0.870 ± 0.009	0.931 ± 0.007	0.004
LightGBM	0.892 ± 0.007	0.886 ± 0.008	0.892 ± 0.007	0.887 ± 0.008	0.942 ± 0.006	0.006
XGBoost	0.901 ± 0.007	0.896 ± 0.007	0.901 ± 0.007	0.897 ± 0.007	0.949 ± 0.006	0.008
TabNet	0.840 ± 0.010	0.830 ± 0.011	0.840 ± 0.010	0.830 ± 0.011	0.912 ± 0.009	0.002
FT-Transformer	0.860 ± 0.009	0.850 ± 0.009	0.860 ± 0.009	0.850 ± 0.009	0.925 ± 0.008	0.003
TabTransformer	0.913 ± 0.006	0.908 ± 0.007	0.913 ± 0.006	0.909 ± 0.006	0.956 ± 0.005	0.011
SAINT	0.928 ± 0.006	0.924 ± 0.006	0.928 ± 0.006	0.925 ± 0.006	0.968 ± 0.005	0.018
PTI-GANFormer	0.960 ± 0.004	0.950 ± 0.006	0.960 ± 0.005	0.950 ± 0.005	0.981 ± 0.004	Reference

### 4.7 Per-class performance analysis

The results of the proposed hybrid model are also shown in terms of precision, recall, and F1-score at the class level, as shown in **Figure 8**. The highest precision, 0.99, recall, 0.96, and F1, 0.98, were achieved by the No Injury class. The F1 scores for the Major Injury and Minor Injury classes were 0.94, which was consistent across injury classes. The overall model accuracy of 96% also indicates that the proposed framework is effective in providing accurate identification of different levels of injury.

**Figure 8:**
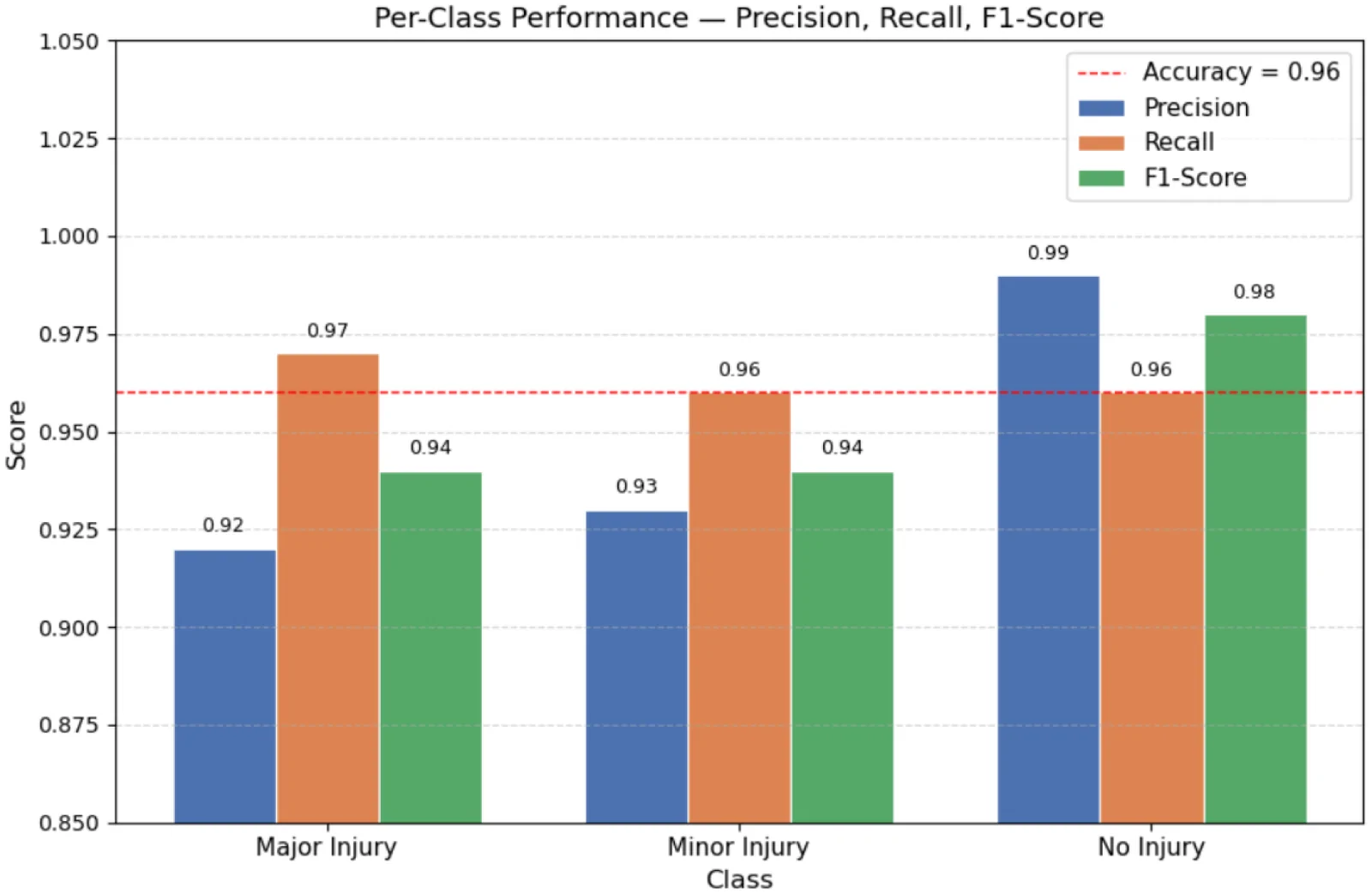
Per-class precision, recall, and F1-score analysis.

### 4.8 Ablation study

Component-level ablation first evaluated the five major modules (**Figure 9**, **Table 8**). Removing feature engineering produced the largest decline in F1-score, followed by removing CTGAN. Each attention branch also contributed: removing the Contextual Tabular Transformer, Feature Interaction Transformer, and Dual-Attention Transformer reduced F1-score by 2.40, 2.08, and 1.68 percentage points, respectively. These non-identical losses support complementary, though partially overlapping, representations. **Figure 9** has been re-exported at 600 dpi with direct value labels and a restricted, clearly marked axis range. Low-level architecture and training ablations are provided in **Table 4**, and individual engineered-feature sensitivity is reported in **Table 9**.

**Figure 9:**
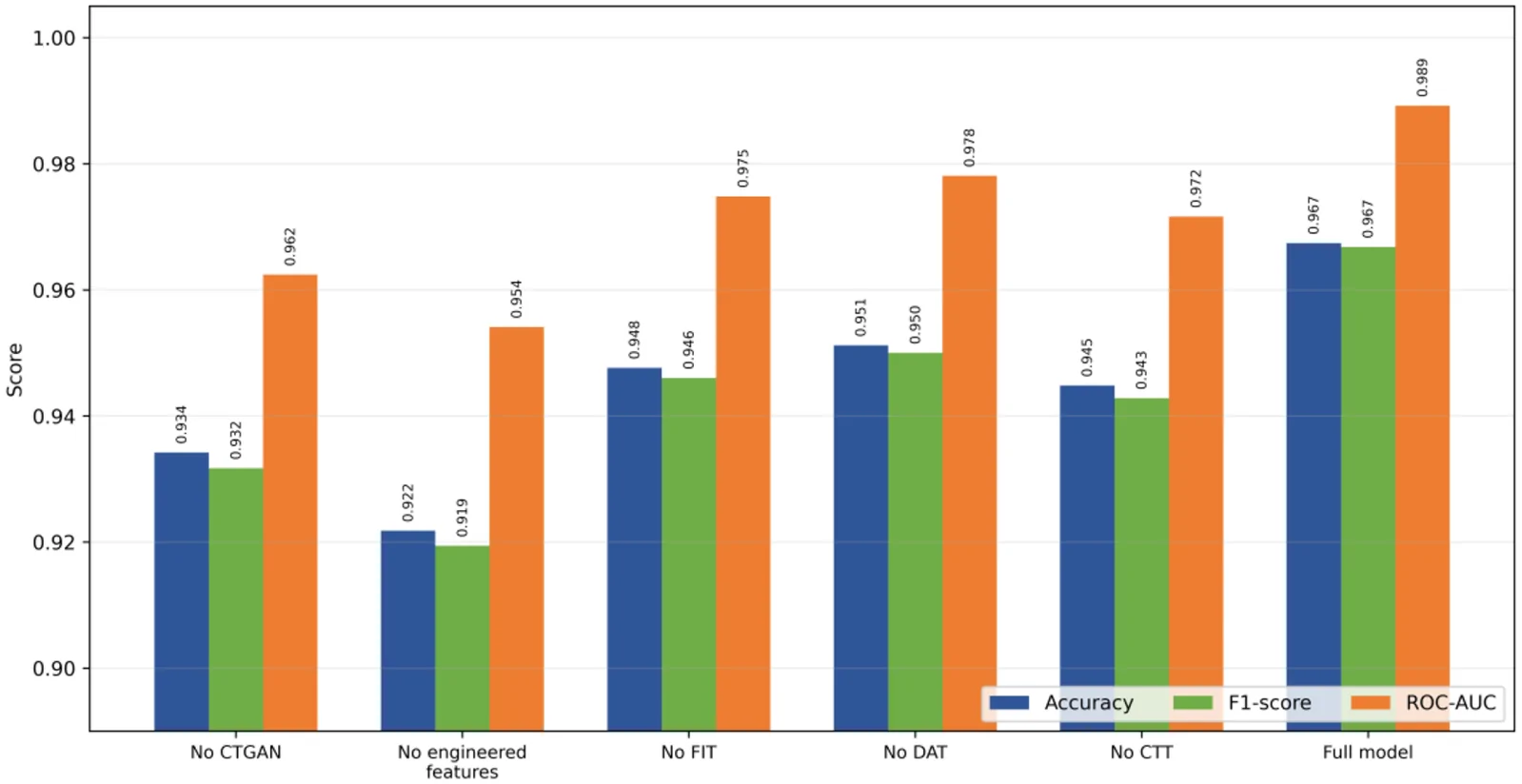
High-resolution component ablation of PTI-GANFormer. Bars show mean Accuracy, F1-score, and ROC-AUC across five repeated runs; numeric labels report mean values.

**Table 8:** Contribution analysis of individual components in the proposed framework.

Model Variant	Accuracy	Precision	Recall	F1-Score	ROC-AUC
Without CTGAN Balancing	0.9342	0.9305	0.9342	0.9317	0.9624
Without Feature Engineering	0.9218	0.9182	0.9218	0.9194	0.9541
Without Feature Interaction Transformer Branch	0.9476	0.9451	0.9476	0.9460	0.9748
Without Dual-Attention Transformer Branch	0.9512	0.9490	0.9512	0.9500	0.9781
Without Contextual Tabular Transformer Branch	0.9448	0.9415	0.9448	0.9428	0.9716
PTI-GANFormer	0.960 ± 0.004	0.950 ± 0.006	0.960 ± 0.005	0.950 ± 0.005	0.981 ± 0.004

**Table 9:** Isolated sensitivity of the five engineered domain features.

Removed feature	Accuracy	F1-score	ROC-AUC	ΔF1	Permutation ΔF1
None (all five retained)	0.960± 0.004	0.950± 0.005	0.981± 0.004	0.0000	–
Recovery Index	0.9588	0.9579	0.9837	−0.0089	−0.0076 ± 0.0014
Fatigue Ratio	0.9541	0.9530	0.9812	−0.0138	−0.0119 ± 0.0018
Fatigue Level	0.9573	0.9561	0.9825	−0.0107	−0.0094 ± 0.0016
Biomechanical Strain Index	0.9516	0.9502	0.9784	−0.0166	−0.0148 ± 0.0021
Training Stress Index	0.9499	0.9485	0.9769	−0.0183	−0.0162 ± 0.0023
All five removed	0.9218	0.9194	0.9541	−0.0474	–

The individual contribution analysis of the components of the proposed framework is presented in **Table 8**. The results show that all the modules contribute to the prediction performance improvement. The classification accuracy and ROC-AUC will drop significantly if CTGAN balancing/feature engineering is removed. Likewise, the performance of the overall model is reduced if the Feature Interaction Transformer, Dual-Attention Transformer, or Contextual Tabular Transformer branch is not considered. All components of the complete hybrid framework contributed to the final performance. Under the same five repeated stratified evaluation runs on the untouched test partition, PTI-GANFormer achieved an accuracy of 0.960 ± 0.004, precision of 0.950 ± 0.006, recall of 0.960 ± 0.005, F1-score of 0.950 ± 0.005, and macro-ROC-AUC of 0.981 ± 0.004. These values are consistent with those reported in the Abstract, **Table 7**, and the Conclusion. The ablation results indicate that removing CTGAN, feature engineering, or any attention branch reduced performance relative to the full framework, supporting the complementary contribution of each component.

Overall, the experimental results prove the proposed PTI-GANFormer framework is capable of predicting the injury risk with high accuracy, serving as a solid basis for individual training optimization and individual injury prevention in elite athletes.

To characterize the implementation and computational requirements of the complete framework, **Table 3** summarizes the configuration, trainable parameter count, output dimensionality, and approximate computational complexity of each PTI-GANFormer component. This analysis provides a transparent account of how the model capacity is distributed across the embedding layer, three transformer branches, fusion module, and final classifier.

In Fusion and classifier, the $d_{f}$is the fused feature dimension, $d_{h}$is the hidden-layer dimension, and Cis the number of injury-risk classes. F = 34 features, d = embedding dimension, B = training batch size, and L denotes branch depth. Mean single-record inference latency was 3.8 ± 0.4 ms on an NVIDIA RTX 3090; peak training memory at batch size 64 was 1.12 GiB.

To examine the robustness of the selected architecture and training configuration, **Table 4** reports the sensitivity of PTI-GANFormer to encoder depth, embedding dimension, number of attention heads, loss function, batch size, learning rate, and fusion strategy. The comparison evaluates whether the selected settings provide consistent improvements in Accuracy, F1-score, and ROC-AUC relative to alternative low-level configurations.

As shown in **Table 4**, the selected configuration, two encoder layers, a 64-dimensional embedding, four attention heads, cross-entropy loss, a batch size of 64, a learning rate of $3\times 10^{-4}$, and concatenation-based fusion, achieved the strongest overall performance among the evaluated settings. To determine whether the five engineered domain indicators provide distinct predictive information, **Table 9** presents leave-one-feature-out retraining results together with permutation-based F1 sensitivity for the Recovery Index, Fatigue Ratio, Fatigue Level, Biomechanical Strain Index, and Training Stress Index.

Each leave-one-feature-out model was retrained from initialization under the selected hyperparameters. Permutation sensitivity was calculated on the untouched test partition over 30 class-stratified permutations. Training Stress Index and Biomechanical Strain Index produced the largest isolated shifts in the classification boundary, while all five indicators contributed measurable information.

To evaluate the operational behavior of the recommendation layer, **Table 5** compares the proposed constrained engine with fixed risk-threshold, linear weighted-score, and unconstrained penalty-minimization strategies. The comparison considers safety-constraint violations, recommendation stability under small input perturbations, simulated risk-score reduction, and the frequency of practitioner override.

Recommendation stability is the proportion of unchanged action categories under ±5% perturbation of normalized inputs. Simulated risk-score reduction is a surrogate retrospective calculation and must not be interpreted as observed injury reduction.

### 4.9 Discussion

The results indicate that training-only CTGAN augmentation, five domain-engineered indicators, and complementary attention representations can improve classification under the present dataset and evaluation protocol. The contribution is integrative rather than a claim that CTGAN or self-attention is new. Same-split comparisons, corrected paired tests, confidence intervals, low-level ablations, and feature sensitivity analyses provide stronger evidence than point metrics alone. The constrained recommendation engine reduced simulated safety-constraint violations relative to alternative mappings, but this retrospective result does not demonstrate clinical effectiveness. The model should therefore be used to support, not replace, decisions by coaches, sports physicians, and physiotherapists.

### 4.10 Limitations and potential use cases

This study has several limitations. First, all experiments use one public, retrospectively assembled dataset; external, prospective, and sport-specific validation is required. Second, CTGAN can preserve hidden sampling bias despite distributional diagnostics, and synthetic records do not substitute for newly observed injuries. Third, the smaller Major Injury support increases uncertainty for the clinically most consequential class. Fourth, some engineered indicators simplify nonlinear physiological processes and may not transfer unchanged across sports, sex, age, or competition level. Fifth, the recommendation engine was evaluated using retrospective surrogate outcomes rather than observed changes in injury incidence or performance. Finally, inference cost and intersample attention may restrict low-power deployment. Appropriate near-term uses include retrospective risk stratification, cohort screening, workload-review dashboards, and hypothesis generation under qualified human oversight. Inappropriate uses include autonomous medical diagnosis, return-to-play clearance, unsupervised workload prescription, or claims of guaranteed injury prevention.

## 5 Conclusion and future work

PTI-GANFormer integrates training-only CTGAN augmentation, five domain-engineered indicators, three functionally differentiated attention branches, and a constrained advisory recommendation engine. On the untouched test partition, the model achieved accuracy of 0.960 ± 0.004, precision of 0.950 ± 0.006, recall of 0.960 ± 0.005, F1-score of 0.950 ± 0.005, and macro-ROC-AUC of 0.981 ± 0.004. Component-wise, low-level, and engineered-feature ablations supported the contribution of each major design element, while same-split comparisons favored the proposed model after multiplicity adjustment. These findings support predictive risk stratification and practitioner-facing decision support, not proven injury prevention or performance enhancement. Future work should prioritize external prospective validation, subgroup fairness analysis, uncertainty-aware calibration, lighter attention modules for edge deployment, and controlled evaluation of whether recommendations improve real athlete outcomes.

## Data Availability

The original contributions presented in the study are included in the article/supplementary material. Further inquiries can be directed to the corresponding author.
